# Wind Turbine Gearbox Condition Monitoring Based on Class of Support Vector Regression Models and Residual Analysis

**DOI:** 10.3390/s20236742

**Published:** 2020-11-25

**Authors:** Harsh S. Dhiman, Dipankar Deb, James Carroll, Vlad Muresan, Mihaela-Ligia Unguresan

**Affiliations:** 1 Department of Electrical Engineering, Adani Institute of Infrastructure Engineering, Ahmedabad 382421, India; harsh.dhiman@aii.ac.in; 2 Department of Electrical Engineering, Institute of Infrastructure Technology Research and Management, Ahmedabad 380026, India; 3 Department of Electronics and Electrical Engineering, University of Strathclyde, Glasgow G1 1XQ, UK; j.carroll@strath.ac.uk; 4 Department of Automation, Technical University of Cluj-Napoca, 400114 Cluj-Napoca, Romania; vlad.muresan@aut.utcluj.ro; 5 Department of Chemistry, Technical University of Cluj-Napoca, 400114 Cluj-Napoca, Romania; Mihaela.Unguresan@chem.utcluj.ro

**Keywords:** condition monitoring, wind turbines, support vector regression, SCADA, neural network, neighborhood component analysis, residual analysis

## Abstract

The intelligent condition monitoring of wind turbines reduces their downtime and increases reliability. In this manuscript, a feature selection-based methodology that essentially works on regression models is used for identifying faulty scenarios. Supervisory control and data acquisition (SCADA) data with 1009 samples from one year and one month before failure are considered. Gearbox oil and bearing temperatures are treated as target variables with all the other variables used for the prediction model. Neighborhood component analysis (NCA) as a feature selection technique is employed to select the best features and prediction performance for several machine learning regression models is assessed. The results reveal that twin support vector regression (99.91%) and decision trees (98.74%) yield the highest accuracy for gearbox oil and bearing temperatures respectively. It is observed that NCA increases the accuracy and thus reliability of the condition monitoring system. Furthermore, the residuals from the class of support vector regression (SVR) models are tested from a statistical point of view. Diebold–Mariano and Durbin–Watson tests are carried out to establish the robustness of the tested models.

## 1. Introduction

Growing energy demands globally have raised concerns leading stakeholders towards renewable energy [1,2]. Industrial development in developing countries has called upon the need to increase the installed capacity. In the past decade, it has been found that wind farms can serve both purposes of increasing installed capacity and minimising environmental concerns. However, with increased installation, more turbine failures occur, thereby driving the need for investment and research in condition monitoring (CM) systems [3,4]. Wind turbines observe highly irregular loads due to stochastic and turbulent wind conditions, which makes components undergo high stress throughout their lifetime [5]. With increasing offshore wind farm installations, the operation and maintenance (O+M) cost is identified as an area with cost-saving potential [6]. Literature suggests that O+M costs for offshore wind turbines can be up to 30% of the overall cost of energy [7]. A major part of CM involves identifying patterns in wind turbine variables like rotor speed, power output, gearbox temperature, gear bearing temperature and generator faults. A commonly used CM system in modern-day wind turbines consists of sensor-based networks that create and record data streams from which the failure is identifiable. Often, the prognosis of wind turbine components is carried out by segmenting the same into different subsystems, such as mechanical and electrical components. Basic CM techniques include vibration analysis (wheels and bearings of the gearbox, generator bearings), oil analysis, thermography and acoustic monitoring (sensors mounted on the turbine equipment) [8]. Nondestructive testing (NDT) is a common way to evaluate the structural integrity of several underground structures [9,10] and aerospace applications [11]. Furthermore, integration of sensors and data mining based technologies has enabled a reliable monitoring process [12].

Gill et al. studied condition monitoring based on a modeling wind turbine power curve where an anomalous behavior in a wind turbine is identified from the deviation-caused turbine power [13]. Butler et al. discussed a similar approach based on Gaussian process models to model the wind turbine power curve [14]. Results revealed a performance degradation three months before a failure in the turbine main bearing. Studies have shown that gearbox bearing causes approximately 70% and 50% of turbine downtime for small and medium-scale generators and large-scale generators, respectively. Availability of gearbox bearing, oil, nacelle temperature through supervisory control and data acquisition (SCADA) has emerged as a popular candidate among the research community to facilitate data-based preventive maintenance for wind turbines. SCADA data are transmitted and stored at an averages of 10 min, which makes the processing speed and storage much easier for the operator. As far as fault identification and diagnosis are concerned, machine learning techniques are often classified in two ways, that is, classification and regression-based approaches. Since machine learning classification problems are based on the prediction of a discrete variable, the turbine condition is classifiable into “healthy” or “abnormal” states, and algorithms used for classification are assessed based on metrics such as accuracy, precision, recall and F1-score, while regression-based approaches are based on the prediction of a continuous variable. The predicted variable is compared against the measured one and error metrics such as mean squared error and mean absolute error are used to compare model effectiveness.

The literature on the classification and regression-based condition monitoring of wind turbines is discussed. Leahy et al. presented a support vector machine (SVM)-based fault diagnosis and fault classification using SCADA data for a 3 MW turbine located in Ireland [15]. A total of 29 features are used to train the SVM algorithm. Results reveal an accuracy of 80% with a recall value in the range of 78–95%. Ibrahim et al. presented a neural network-based model for the detection of mechanical faults in a wind turbine [16]. The model is based on a current signal acquired at different ranges of speed, used as an input with classification accuracy in the range of 93–98%. In [17], authors implement Shannon wavelet-based SVM technique for the fault classification of wind turbine gearbox faults. A non-linear feature selection technique is used with SVM resulting in an accuracy of 92% compared to 72% with standard SVM with radial basis function as a kernel. Jiang et al. studied a multi-scale convolutional neural network (CNN) for fault diagnosis of a wind turbine [18]. In this method, the feature extraction for classification task is carried out from the vibration signals. The performance of multi-scale CNN is compared with the conventional CNN method which results in an accuracy of 98.53%. Jiminez et al. studied linear and non-linear feature selection techniques for ice detection in wind turbines [19]. Among linear feature selection techniques, auto-regressive and principle component analysis (PCA) is used, and in case of non-linear feature selection techniques, neighborhood component analysis (NCA) and hierarchical non-linear PCA is used. Ice detection and diagnosis are carried out based on SVM, decision tree, k-nearest neighbors (kNN) and discriminant analysis. In [20], a deep neural network-based technique is applied for anomaly and fault detection in wind turbine components. Based on SCADA data, a deep auto-encoder based deep neural network is established. Carroll et al. discussed logistic regression, two-class neural networks and SVM for classifying gear tooth and gear bearing scenarios from SCADA data [21]. In [22], authors use signal processing techniques such as sideband analysis, time-synchronous analysis, amplitude modulation to extract features for classifying the healthy and abnormal state of a wind turbine. Algorithms like kNN, SVM and decision tree are utilized to achieve the same with an accuracy of 90.2%, 91.3% and 91% respectively.

Anomaly detection can be seen as a problem of both, supervised and unsupervised machine learning [23]. While a majority of anomaly detection problems have been addressed using unsupervised learning such as K-means clustering and local outlier factor (LOF), that estimates the distance between all the samples using K-nearest neighbor concept and deviation in density function [24]. Japkowicz et al. [25] presented novelty detection using a classification approach. In the classification-based approach, each sample in the training set has a labelled output for which the anomalies are obtained. However, with a classification-based approach, the problem of imbalanced instances may arise which deteriorates the quality of the classification model for unseen testing samples. On the other hand, semi-supervised learning assumes that labelled instances are available for normal or healthy classes. In the wind industry, the concept of anomaly detection is utilized to identify vulnerable equipment in the machine using a data mining approach [26,27].

The motivation behind this work arises from the ability of a class of SVR models to yield excellent results in the case of a wind speed forecasting scenario [28]. SVR models with a particular choice of loss function result in an optimal estimate for a given noise model. For example, a quadratic loss function in the least square support vector regression (LSSVR) model enables it to perform optimally for a normally distributed noise. While a majority of the work on wind turbine condition monitoring is reported as a classification task to the best of our knowledge, we are the first group to use a class of SVR models and discuss residual analysis for gearbox condition monitoring. The main contributions of this work are as follows:

1. Predictive analytics for wind turbine gearbox are studied based on a class of support vector regression (SVR) models in the form of twin support vector regression combined with neighborhood component analysis. The impact of feature selection is studied on the prediction metrics of gearbox oil and bearing temperature.
2. The SCADA data procured consist of a list of variables that are scanned under the banner of feature selection for the accurate prediction of gearbox oil and bearing temperature. The performance of SVR based models as compared to the multi-layer perceptron neural network, decision tree and logistic regression, is presented.
3. Statistical analysis is carried out for SVR based models to analyze residuals and their correlation. Specifically, Diebold–Mariano and Durbin–Watson tests help analyze the residuals for establishing the robustness among tested models.

The organization of this manuscript is as follows: Section 2 gives an idea about the machine learning-based regression methods considered in this analysis. Methods like SVR and its variants, neural network, decision tree and logistic regression are presented with their mathematical formulation and parameters involved. Furthermore, in Section 2, the feature selection technique based on neighborhood component analysis is discussed with a graphical illustration. Section 3 consists of the description of SCADA data where various feature variables are highlighted; the experimental results concerning the prediction analysis are discussed followed by Conclusions in Section 4.

## 2. SVM-Based Data-Driven Models

Given the training set $T=\{\left(x_{i},y_{i}\right):x_{i}\in R^{n},y_{i}\in R,\;i=1,2\ldots ,l\;\}$, with *l* samples, support vector regression (SVR) models minimize a linear combination of the loss function and regularization term for obtaining its linear estimate $f\left(x\right):w^{T}x+b,\;w\in R^{n},b\in R$. For estimating the non-linear function, it will find $f\left(x\right):K\left(x^{T},A^{T}\right)u+b$ in feature space, where *K* is the appropriate kernel satisfying Mercer condition [29].

### 2.1. ϵ-Support Vector Regression Model

The ϵ-SVR model minimizes the ϵ-insensitive loss function along with the $\frac{1}{2}w^{T}w$ regularization. It finds the solution of the following optimization problem

(1) $$\min_{w,b}\frac{1}{2}w^{T}w+C\sum_{i=1}^{l}{\left|y_{i}-f\left(x_{i}\right)\right|}_{\epsilon },$$

where $|y_{i}-f\left(x_{i}\right){|}_{\epsilon }=$ max$\left(0,|y_{i}-f\left(x_{i}\right)|-\epsilon \right)$ is the ϵ-insensitive loss function which can ignore an error up to ϵ. After introducing the slack variable $\kappa_{i}$ and $\kappa_{i}^{*}$ for i=1,2,…,l, the ϵ-SVR problem (1) is solved by converting the QPP:

(2) $$\begin{array}{cc} \min_{w,b,\kappa ,\kappa^{*}}\;\;\frac{1}{2}{∥w∥}^{2}+C\sum_{i=1}^{l}\left(\kappa_{i}+\kappa_{i}^{*}\right)\\ & \;\mathrm{subject}\;\mathrm{to},\\ & \;y_{i}-\left(A_{i}w+b\right)\leq \epsilon +\kappa_{i},\\ \left(A_{i}w+b\right)-y_{i}\leq \epsilon +\kappa_{i}^{*},\;\kappa_{i},\kappa_{i}^{*}\geq 0. \end{array}$$

### 2.2. Least Squares Support Vector Regression model

The LSSVR model [30] minimizes the quadratic loss function along with the $\frac{1}{2}w^{T}w$ regularization term. It minimizes

(3) $$\min_{w,b}\frac{1}{2}w^{T}w+C\sum_{i=1}^{l}{\left(y_{i}-f\left(x_{i}\right)\right)}^{2},$$

in its optimization problem along with the regularization term $\frac{1}{2}{\left||w|\right|}^{2}$. The optimization problem of the LSSVR model can be expressed as

(4) $$\begin{array}{cc} \min_{w,b,\xi }\;\;\frac{c}{2}{∥w∥}^{2}+C_{1}\sum_{i=1}^{l}\left(\xi_{i}^{2}\right)\\ & \;\mathrm{subject}\;\mathrm{to},\;\;y_{i}-\left(A_{i}w+b\right)=\xi_{i},\;\;i=1,2,\ldots ,l, \end{array}$$

where $C_{1}>0$ is a user-defined parameter. The solution of problem (4) can be obtained by solving the system of equations.

### 2.3. Huber Support Vector Regression Model

Huber SVR uses a well defined Huber loss function to solve the regression problem.

(5) $$\min_{w}\frac{1}{2}w^{T}w+C\sum_{i=1}^{l}L_{\mathrm{Huber}}\left(y_{i}-f\left(x_{i}\right)\right)$$

subject to

(6) $$\begin{array}{c} y_{i}-\left(A_{i}w+b\right)\leq \epsilon +\kappa_{1}^{i},\left(i=1,2,\ldots ,l\right)\\ \left(A_{i}w+b\right)-y_{i}\leq \epsilon +\kappa_{2}^{i},\left(i=1,2,\ldots ,l\right)\\ \kappa_{1}^{i}\geq 0,\kappa_{2}^{i}\geq 0,\left(i=1,2,\ldots ,l\right) \end{array}$$

where *C* is the regularization term to improve the trade-off between training error and flatness of the regressor.

(7) $$L_{\mathrm{Huber}}\left(t\right)=\left\{\begin{array}{cc} \frac{1}{2}{\left(t\right)}^{2}, & \;\mathrm{if}\;\;|t|<c\\ c\left(\left|t\right|-\frac{c}{2}\right), & \;\mathrm{other}\;\mathrm{wise}\; \end{array}\right.$$

### 2.4. Twin Support Vector Regression (TSVR)

Twin support vector regression (TSVR) works on the lines of twin support vector machines (TSVM) [31]. TSVR estimates two non-parallel hyperplanes by solving two quadratic programming problems (QPP), thereby reducing the computation effort. Mathematically, these QPPs can be expressed as

(8) $$\begin{array}{c} \min\;\frac{1}{2}{\left(Y-e\varepsilon_{1}-\left(Aw_{1}+eb_{1}\right)\right)}^{T}\left(Y-e\varepsilon_{1}-\left(Aw_{1}+eb_{1}\right)\right)\\ +C_{1}e^{T}\kappa \\ s.t.\;Y-\left(Aw_{1}+eb_{1}\right)\geq e\varepsilon_{1}-\kappa ,\kappa \geq 0 \end{array}$$

(9) $$\begin{array}{c} \min\;\frac{1}{2}{\left(Y-e\varepsilon_{2}-\left(Aw_{2}+eb_{2}\right)\right)}^{T}\left(Y-e\varepsilon_{2}-\left(Aw_{2}+eb_{2}\right)\right)\\ +C_{2}e^{T}\upsilon \\ s.t.\;\left(Aw_{2}+eb_{2}\right)-Y\geq e\varepsilon_{2}-\upsilon ,\upsilon \geq 0, \end{array}$$

where $C_{1},C_{2}>0$, $\varepsilon_{1},\varepsilon_{2}\geq 0$ are parameters and κ,υ represent the slack variables. A detailed explanation of optimization problem and KKT conditions can be found in [31]. In case of non-linear regression, kernel technique can be used to solve the QPP. The kernel-based TSVR estimates the mean of the two regressors $g_{1}\left(x\right)=K\left(x^{T},A^{T}\right)w_{1}+b_{1}$ and $g_{2}\left(x\right)=K\left(x^{T},A^{T}\right)w_{2}+b_{2}$.

### 2.5. Neighborhood Component Analysis

Modern day tasks such as classification, clustering, regression and pattern recognition work with a significant amount of data in order to trace a meaningful relationship. High volumes of data in terms of features can lead to the problem of over-fitting. To deal with such scenarios, neighborhood component analysis (NCA) works on the principle of distance learning analogous to K-nearest neighbors (kNN). The aim is to determine relevant/important features corresponding to the variable of interest (target variable) [32]. Given a set of input training examples $X=\{x_{1},x_{2},\ldots ,x_{n}\}$ and $Y=\{y_{1},y_{2},\ldots ,y_{n}\}$ being the target variable, NCA estimates a projection matrix *S* with dimension r×d, where $B=S^{T}S$ computes the training data in a *r*-dimension space. The elements of the projection matrix *S* can be expressed as

(10) $$p\left(x_{i},x_{j}\right)={\left(Sx_{i}-Sx_{j}\right)}^{T}\left(Sx_{i}-Sx_{j}\right),$$

where *p* presents a distance metric given $x_{i}$ and $x_{j}$ as training and testing samples. In case of NCA, a non-convex optimization problem is solved with marked labels being used to determine close neighbors, which is not the case with PCA. Consider $h_{ij}$ to be the probability of locating a label *j* close to its neighbor *i* as

(11) $$h_{ij}=\frac{\exp(-\|Sx_{i}-Sx_{j}{\|}^{2})}{\sum_{k\neq i}\exp(-\|Sx_{i}-Sx_{j}{\|}^{2})}.$$

**Figure 1:**
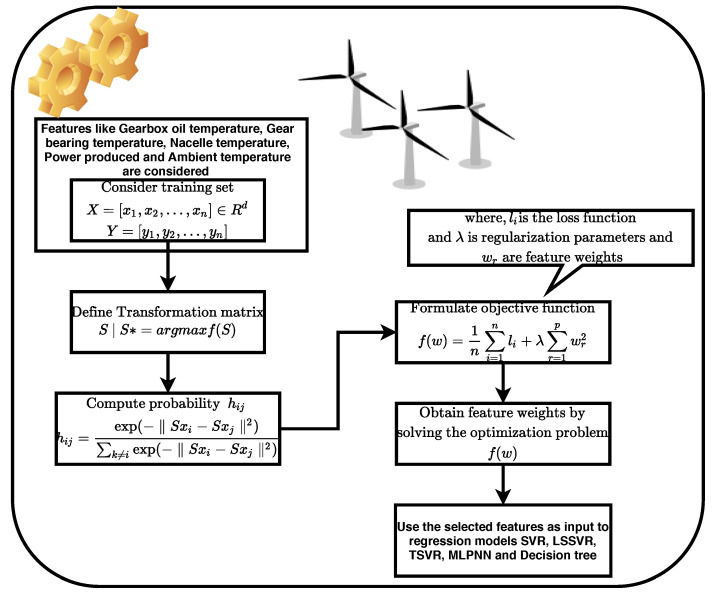
Neighborhood component analysis (NCA) algorithm for feature selection in wind turbine gearbox condition monitoring.

Furthermore, a correct classification of neighbors can be expressed in terms of an optimization function f(S) as

(12) $$\begin{array}{ccc} f(S) & = & \sum_{i}\sum_{j\in C_{i}}h_{ij}=\sum_{i}h_{i} \end{array}$$

(13) $$\begin{array}{ccc} \frac{\partial f}{\partial S} & = & -2S\sum_{i}\sum_{j\in C_{i}}h_{ij}\left(x_{ij}x_{ij}^{⊤}-\sum_{k}h_{ik}x_{ik}x_{ik}^{⊤}\right), \end{array}$$

where $x_{ij}=x_{i}-x_{j}$. Figure 1 depicts a flowchart for weight estimation of features based on NCA.

In the figure, $l_{i}$ represents a loss function which can be expressed in terms of mean absolute deviation. With respect to the current work, variables acquired from SCADA data are considered for feature selection which are fed as an input to the regression models. Some of the variables are depicted in Table 1. In the next Section, the performance parameters from regression models are discussed considering feature selection based on NCA.

## 3. Results and Discussion

**Figure 2:**
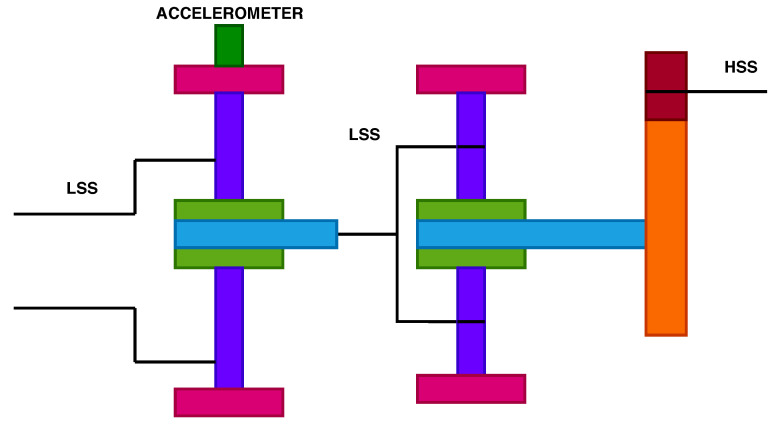
Gearbox type “A” configuration and location of accelerometer.

**Figure 3:**
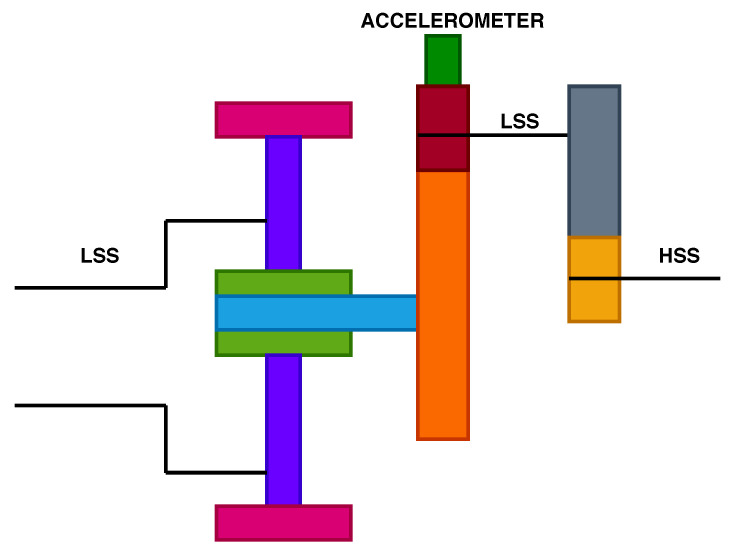
Gearbox type “B” configuration and location of accelerometer.

According to the National Renewable Energy Laboratory’s (NREL) Gearbox Reliability Database (GRD), 76% of gearboxes failures happen due to bearings, and 17% from gear failures [33]. The gearbox models include a low-speed planetary stage (LSS), an intermediate stage and a high-speed parallel stage (HSS) as illustrated in Figure 2 and Figure 3. The two gearbox models come from a different turbine type. The gearbox configurations are used extensively for turbines rated 2 MW and 4MW, with rotor diameter ranging between 80 to 120 m. Both turbine types use high-speed gearboxes with induction generators. Gearbox “A” consists of two planetary stages and one parallel stage, while gearbox type “B” consists of one planetary stage and two parallel stages.

**Figure 4:**
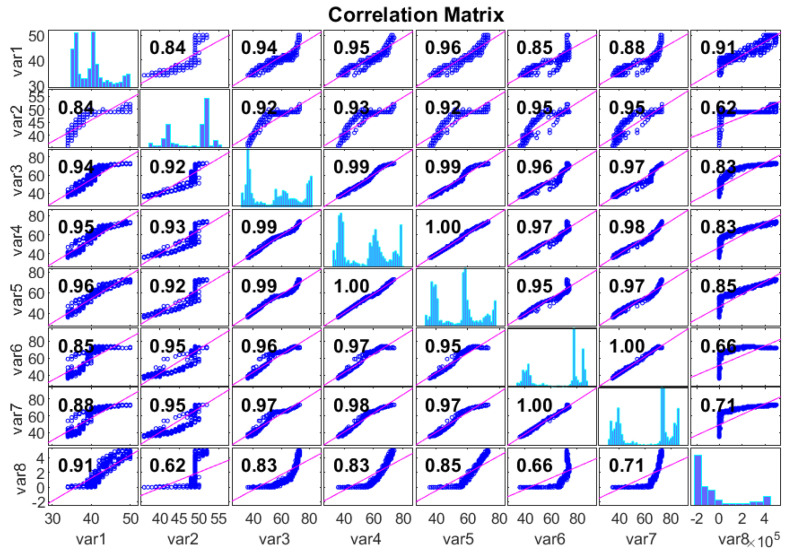
Pearson correlation plot for supervisory control and data acquisition (SCADA) input variables.

Figure 4 describes the correlation coefficient between gearbox oil and the bearing temperature. Table 1 depicts the computations of the Pearson correlation coefficient for these variables in this analysis. The sampling interval of these variables is 10 min, which is appropriate from an industrial standpoint, as the majority of the market-clearing operations are taking place in this interval. The data acquired are checked for missing values. After corroboration of the SCADA data quality, the sensor temperature readings are subtracted from each other to obtain differences in temperature (ΔT) to train the supervised learning algorithms. For example, as acquired from SCADA data, a gear oil temperature reading ($T_{oil}$) and an ambient temperature reading ($T_{amb}$) are used to determine the difference in temperatures as $\Delta T=T_{oil}-T_{amb}$. The ΔT provide extra variables for training and testing.

The experimental setup is as follows; all experiments have been performed on Intel Core i3 6th generation processor with 4 GB of RAM in MATLAB 18.0 environment (http://in.mathworks.com/). In this section, we discuss the experimental results for the predictive analytics performed on a wind turbine. SCADA data available for 1 year and 1 month prior to failure consist of several variables, as discussed in the previous section. Gearbox oil and bearing temperature are analyzed through a regression-based approach. Since the temperature of oil and bearing is a continuous variable, a regression-based approach helps one to identify abnormal trends in its time-series. Since the available SCADA consists of 54 feature variables (27 from 1 year prior and rest from 1 month before failure), it is important to identify important features and remove redundant ones. One such feature selection technique is the neighborhood component analysis as described in Section 2. We choose the best-suited features as input to the machine learning-based regression model. In this manuscript, the prediction of the gearbox oil (sensor 1) and bearing temperatures takes place, using a set of ML techniques. In Table 2, variable index 1 is treated as target variable, and the rest of the 53 (26 + 27) variables are considered inputs to the model. It is important to note that machine learning algorithms work well with a significant amount of data, and hence getting the right amount of data from SCADA systems is essential for identifying faulty situations. SCADA data for this analysis consist of 1009 samples, out of which 800 samples are used in the training phase and rest for testing.

**Figure 5:**
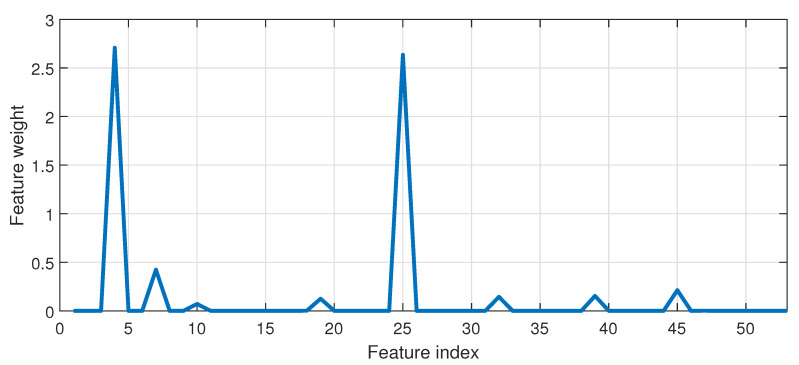
Weights based on NCA for corresponding feature variables.

Figure 5 illustrates the weights corresponding to the feature variables.

A detailed description of SCADA variables can be found in Table 2. It is observed that feature variables such as ΔT oil sensor 1 and ambient, gear bearing 1 temperature, ambient temperature and nacelle temperature have significant weightage compared to others. Hence, in order to predict gearbox oil temperature, these variables are treated as features or inputs to the prediction model.

**Figure 6:**
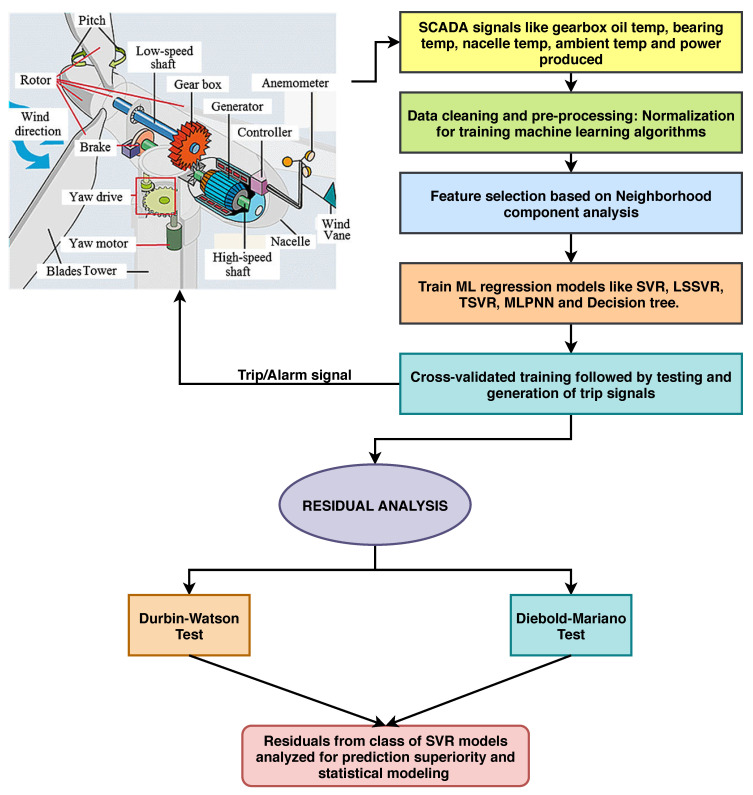
Flowchart for wind turbine gearbox condition monitoring via class of support vector regression (SVR) models.

The detailed flowchart of the proposed methodology is illustrated in Figure 6. Since machine learning models are stochastic, to validate the importance of training data, we perform 10-fold cross-validation for all models and compute the accuracy results with a standard deviation error, as depicted in Table 3 and Table 4. The bold values indicate the prediction results when NCA is used as a feature selection technique.

Among the tested regression techniques, decision trees give minimum RMSE values. This behavior of decision trees can be understood with its simplicity while predicting unseen data once trained with an optimal number of features. Out of the tested models, for condition monitoring of wind turbine, it is observed that for diagnosing a failure associated with gearbox oil temperature, TSVR gives accurate prediction. However, for raising alarms regarding failures in gearbox bearing, a decision tree-based model yields highest accuracy. This analysis is carried out with SCADA data 1 month and 1 year prior to failure. Reducing the dimension of feature space using NCA reduces computational effort and increases reliability of an intelligent condition monitoring system for wind turbines.

## 4. Residual Analysis

In this section, the residuals from gearbox oil temperature prediction are analyzed from a statistical point of view. Methods like TSVR, LSSVR and Huber-SVR are tested against standard-SVR using a Diebold–Mariano (DM) test that compares the accuracy of two forecasting models. According to a DM test, when two prediction models have similar accuracy, a null hypothesis is adopted [34]. With respect to the current objective, the DM test is conducted with TSVR (Test 1), LSSVR (Test 2) and Huber-SVR (Test 3) to test its accuracy against standard the SVR model. The results are depicted in Table 5 with 1% significance level, and it is observed that, TSVR, LSSVR and Huber-SVR models have substantial prediction edge over a standard SVR (ε-SVR) model, thereby establishing the robustness of the tested models.

Furthermore, in order to examine the nature of residuals and potential auto-correlation among them, the Durbin–Watson (DW) statistic is computed for the class of SVR models. The test is based on the fact that for a statistical regression model, the errors are independent. The DW statistic can be given as

(14) $$DW=\frac{\sum_{i=2}^{n}{\left(e_{i}-e_{i-1}\right)}^{2}}{\sum_{i=1}^{n}e_{i}^{2}},$$

where $e_{i}$ denotes the *i*th error term of a n×1 error column vector. The error vector for class of SVR models is tested for potential autocorrelation which can be modeled in the form of a hypothesis as follows

(15) $$\begin{array}{cc} \; & \;\mathrm{If}\;DW<d_{L}\;\;\mathrm{reject}\;H_{0}:\rho =0\\ \; & \;\mathrm{If}\;DW>d_{U}\;\;\mathrm{do}\;\mathrm{not}\;\mathrm{reject}\;H_{0}:\rho =0\\ \; & \;\mathrm{If}\;d_{L}<DW<d_{U}\;\;\mathrm{test}\;\mathrm{is}\;\mathrm{inconclusive}.\; \end{array}$$

where $d_{L}$ and $d_{U}$ are the lower and upper critical limits which can be found from the DW table for any α-level of significance [35]. In this manuscript, the DW statistic is calculated at 1% significance level and is tabulated in Table 6.

**Figure 7:**
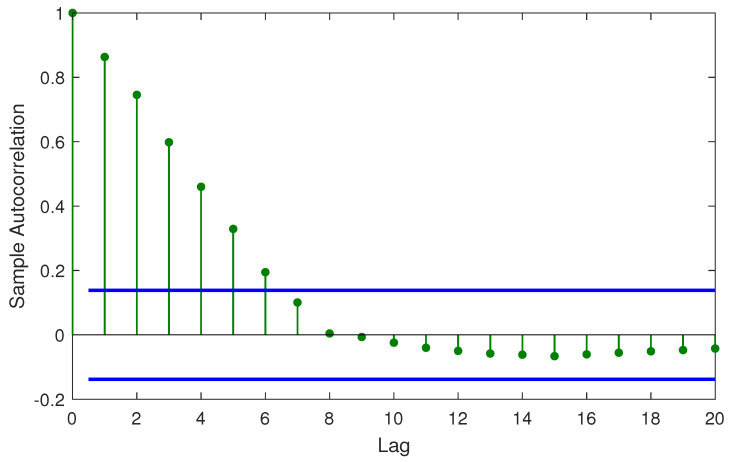
Autocorrelation for residuals of Huber-SVR.

The results of the DW test indicate that for the class of SVR models, the errors are auto-correlated and can be represented by an auto-regressive (AR) process of suitable lag. For example, Figure 7 illustrates the autocorrelation at various lag instants. It is observed that till lag instant 6, the errors indicate a high level of autocorrelation. A typical *p*-order AR process with lag coefficients $\beta_{1},\beta_{2},\ldots ,\beta_{p}$ and noise ϵ can be expressed as follows

(16) $$y_{t}=\beta_{0}+\beta_{1}y_{t-1}+\beta_{2}y_{t-2}+\cdots +\beta_{p}y_{t-p}+\epsilon_{t}.$$

**Figure 8:**
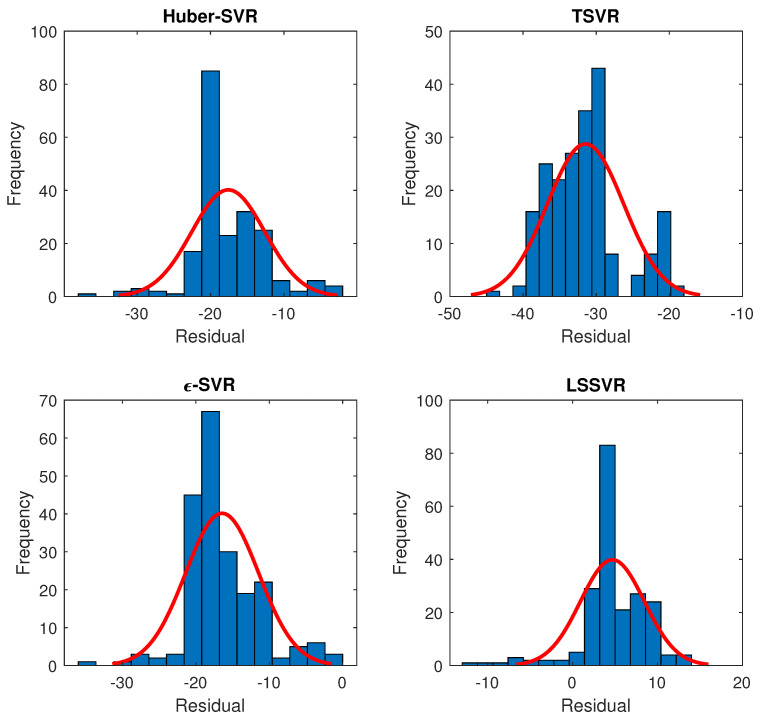
Normal distribution fitting of gearbox temperature residuals from class of SVR models.

The main idea behind carrying out residual analysis is to identify the time-series relationship for a class of SVR models. The residuals obtained from ε-SVR, LSSVR, TSVR and Huber-SVR are tested for the identification of the orders of ARIMA models. Table 7 depicts statistical parameters of ARIMA models, and we observe that LSSVR and TSVR follow ARIMA order (4,1,1) and (3,1,1) respectively. We find that the Akaike information criteria (AIC) and Bayesian information criteria (BIC) values for LSSVR and TSVR are lower as compared to SVR and Huber-SVR, indicating that the fitted ARIMA orders reflect the true model. AIC and BIC of the ARIMA models for residuals are computed in R studio. Figure 8 represents the fitting of residuals obtained from a class of SVR methods. The residuals obey a certain distribution. The majority of the time for wind speed and power forecast errors, the distribution that fits well is Gaussian distribution. It is observed that for residuals of TSVR, the distribution closely follows normal distribution, hence making it feasible to forecast the gearbox oil and bearing temperature with higher accuracy. Regression-based approach for condition monitoring may be further extended to minimize the false positive rate that is a pertinent issue with most of the classification algorithms. Since the gearbox oil and bearing temperature is essentially a time-series, statistical modeling of residuals can help in increasing the turbine reliability in terms of the generation of trip signals. It would also help the operator to schedule the timely maintenance of an unhealthy wind turbine(s). In future, a time-series can be analyzed adaptively to identify instances of anomalous behavior and reduce the false-positive rate which is an issue with classification-based approaches [21].

## 5. Conclusions

This manuscript highlights the importance of feature selection for condition monitoring of wind turbine. Modern day SCADA data contain a lot of variables from which redundant data need to be removed. Neighborhood component analysis for regression aids this process by calculating feature weights. Features with higher importance are considered for regression analysis with methods like SVR, neural network, decision tree and logistic regression under evaluation. Based on the experimental results, we observe that with an accuracy of 99.91 ± 0.007%, a TSVR-based model outperforms all other models for gearbox oil temperature prediction followed by MLPNN. However, for gearbox bearing temperature, decision tree outperforms TVSR, MLPNN and logistic regression with an accuracy of 98.74 ± 0.91%. Quantitatively, with NCA, the prediction accuracy is found superior to without NCA. This is indicative of the fast computation aided by relevant features. From statistical point of view, the residuals are evaluated using the Diebold–Mariano and Durbin–Watson statistics, where the robustness of the tested models is ascertained. For the Durbin–Watson test, ε-SVR, LSSVR, Huber-SVR and TSVR obtain statistic values of 0, 0.284, 0.0234 and 0.0302 respectively, which rejects null hypothesis and indicates the presence of autocorrelation among residuals for tested models. The residuals are also analyzed for their ARIMA orders and it is observed that LSSVR and TSVR depict close relationship in their AIC values of 691.53 and 696.62 respectively. Furthermore, it must be noted that in this study Bayesian analysis is not considered because of the dependency among feature variables.

## Figures and Tables

**Table 1:** SCADA data feature index description.

Variable Index	Particular
var1	Gear oil 1 temperature
var2	Gear oil 2 temperature
var3	Gear bearing 1 temperature
var4	Gear bearing 2 temperature
var5	Gear bearing 3 temperature
var6	Gear bearing 4 temperature
var7	Gear bearing 5 temperature
var8	Total power production

**Table 2:** Detailed description of SCADA data variables.

Variable Index	Particular	Units
1	Gear oil 1 temperature	(${}^{{}^{\circ}}$C)
2	Gear oil 2 temperature	(${}^{{}^{\circ}}$C)
3	ΔT oil sensor 1 and oil sensor 2	(${}^{{}^{\circ}}$C)
4	ΔT oil sensor 1 and nacelle	(${}^{{}^{\circ}}$C)
5	ΔT oil sensor 1 and ambient	(${}^{{}^{\circ}}$C)
6	ΔT oil sensor 2 and nacelle	(${}^{{}^{\circ}}$C)
7	ΔT oil sensor 2 and ambient	(${}^{{}^{\circ}}$C)
8	Gear bearing 1 temperature	(${}^{{}^{\circ}}$C)
9	Gear bearing 2 temperature	(${}^{{}^{\circ}}$C)
10	Gear bearing 3 temperature	(${}^{{}^{\circ}}$C)
11	Gear bearing 4 temperature	(${}^{{}^{\circ}}$C)
12	Gear bearing 5 temperature	(${}^{{}^{\circ}}$C)
13	ΔT bearing 1 and nacelle	(${}^{{}^{\circ}}$C)
14	ΔT bearing 1 and ambient	(${}^{{}^{\circ}}$C)
15	ΔT bearing 2 and nacelle	(${}^{{}^{\circ}}$C)
16	ΔT bearing 2 and ambient	(${}^{{}^{\circ}}$C)
17	ΔT bearing 3 and nacelle	(${}^{{}^{\circ}}$C)
18	ΔT bearing 3 and ambient	(${}^{{}^{\circ}}$C)
19	ΔT bearing 4 and nacelle	(${}^{{}^{\circ}}$C)
20	ΔT bearing 4 and ambient	(${}^{{}^{\circ}}$C)
21	ΔT bearing 5 and nacelle	(${}^{{}^{\circ}}$C)
22	ΔT bearing 5 and ambient	(${}^{{}^{\circ}}$C)
23	Nacelle temperature	(${}^{{}^{\circ}}$C)
24	Rotor speed	(RPM)
25	Wind speed	(m/s)
26	Ambient temperature	(${}^{{}^{\circ}}$C)
27	Total power production	(Watts)

**Table 3:** Performance metrics for Gearbox oil (sensor 1) temperature prediction.

Method	RMSE	MAPE (%)	MAE	% Acc	NMSE
ε-SVR	38.73 ± 12.1	96.06 ± 1.31	38.15 ± 13.5	3.93 ± 2.31	10.60 ± 4.1
	**0.25 ± 1.01**	**0.46 ± 0.13**	**0.17 ± 0.62**	**99.5 ± 0.01**	**2.41 ± 1.52**
LSSVR	3.08 ± 1.2	6.21 ± 2.4	2.54 ± 1.7	93.79 ± 1.41	2.13 ± 0.51
	**0.09 ± 1.01**	**0.17 ± 0.01**	**0.06 ± 0.001**	**99.82 ± 0.05**	**2.06 ± 0.41**
Huber-SVR	5.40 ± 2.41	11.14 ± 4.13	4.76 ± 1.01	88.86 ± 2.14	3.13 ± 1.97
	**4.38 ± 0.91**	**8.24 ± 1.56**	**3.73 ± 0.51**	**91.76 ± 2.67**	**2.76 ± 0.14**
TSVR	3.07 ± 0.71	6.18 ± 1.97	2.44 ± 0.12	93.82 ± 3.17	1.98 ± 0.51
	**0.04 ± 0.067**	**0.08 ± 0.07**	**0.03 ± 0.02**	**99.91 ± 0.007**	**1.91 ± 0.01**
MLPNN	4.70 ± 0.04	8.39 ± 0.19	3.52 ± 0.09	91.60 ± 0.19	1.88 ± 0.45
	**0.18 ± 0.1**	**0.36 ± 0.14**	**0.14 ± 0.06**	**99.70 ± 0.001**	**0.03 ± 0.044**
LR	2.22 ± 6.67	3.48 ± 2.67	1.45 ± 3.2	96.51 ± 1.07	0.42 ± 0.27
	**1.72 ± 3.69**	**2.44 ± 1.56**	**1.00 ± 1.6**	**97.55 ± 0.75**	**0.25 ± 1.67**
DT	0.60 ± 3.14	0.35 ± 2.14	0.84 ± 5.13	99.65 ± 0.002	0.031 ± 2.12
	**0.43 ± 1.57**	**0.36 ± 1.07**	**0.74 ± 2.67**	**99.64 ± 0.001**	**0.016 ± 1.06**

**Table 4:** Performance metrics for Gearbox bearing 1 temperature prediction.

Method	RMSE	MAPE (%)	MAE	% Acc	NMSE
ε-SVR	54.54 ± 22.67	98.55 ± 0.37	54.13 ± 32.17	3.99 ± 1.02	135.8 ± 81.67
	**3.68 ± 1.07**	**5.53 ± 0.93**	**3.01 ± 3.13**	**94.46 ± 0.11**	0.628 ± 1.41
LSSVR	5.91 ± 2.53	9.29 ± 5.07	4.87 ± 2.08	90.70 ± 5.33	1.594 ± 1.69
	**3.67 ± 1.25**	**5.52 ± 2.05**	**3.01 ± 1.04**	**94.47 ± 2.15**	0.62 ± 1.34
Huber-SVR	6.40 ± 1.56	10.14 ± 3.63	5.76 ± 2.67	89.86 ± 3.95	2.13 ± 1.62
	**4.18 ± 0.78**	**8.46 1.81**	**4.03 ± 1.36**	**91.54 ± 1.97**	**2.06 ± 0.81**
TSVR	5.80 ± 1.45	9.13 ± 3.55	4.80 ± 3.44	90.86 ± 4.24	1.53 ± 0.46
	**3.63 ± 0.89**	**5.50 ± 2.59**	**2.99 ± 2.91**	**94.50 ± 3.13**	**0.60 ± 0.31**
MLPNN	7.26 ± 4.56	10.22 ± 1.73	5.92 ± 0.94	89.77 ± 1.73	1.97 ± 0.512
	**2.84 ± 2.23**	**3.54 ± 0.86**	**2.00 ± 0.47**	**96.45 ± 0.83**	**0.05 ± 0.25**
LR	12.4 ± 2.09	18.00 ± 2.09	10.13 ± 1.39	82.00 ± 2.09	2.26 ± 1.40
	**5.25 ± 1.04**	**6.24 ± 0.06**	**3.64 ± 0.43**	**93.75 ± 0.62**	**0.40 ± 0.74**
DT	4.50 ± 5.70	8.53 ± 1.44	5.39 ± 0.765	89.64 ± 1.44	1.65 ± 0.42
	**1.18 ± 2.35**	**1.26 ± 0.79**	**0.72 ± 0.384**	**98.74 ± 0.91**	**0.02 ± 0.35**

**Table 5:** Diebold–Mariano test for wind turbine gearbox temperature prediction.

Residual	DM Statistic		
	Test 1	Test 2	Test 3
Gearbox oil temperature	44.9821	−21.0747	19.2741
Gearbox bearing temperature	34.1874	−20.1813	10.0529

**Table 6:** Durbin–Watson statistic for SVR models.

Model	DW Statistic	Result
ε-SVR	0	$H_{0}$ rejected
LSSVR	0.284	$H_{0}$ rejected
Huber-SVR	0.0234	$H_{0}$ rejected
TSVR	0.0302	$H_{0}$ rejected

**Table 7:** Statistical analysis of residuals from class of SVR models.

	SVR	LSSVR	TSVR	Huber-SVR
ARIMA Order (p,d,q)	1,1,3	4,1,1	3,1,1	1,1,3
AIC	1198.46	691.53	696.62	1199.18
BIC	1215.15	711.55	713.31	1215.87

## Abbreviations

The following abbreviations are used in this manuscript:

ANN	Artificial neural network
CM	Condition monitoring
DT	Decision tree
LSSVR	Least square support vector regression
LR	Logistic regression
NCA	Neighborhood component analysis
NDT	Nondestructive testing
O+M	Operation and maintenance
PCA	Principle component analysis
SCADA	Supervisory control and data acquisition
SVR	Support vector regression
TSVR	Twin support vector regression
